# Research Funding: the Case for a Modified Lottery

**DOI:** 10.1128/mBio.00422-16

**Published:** 2016-04-12

**Authors:** Ferric C. Fang, Arturo Casadevall

**Affiliations:** a Departments of Laboratory Medicine and Microbiology, University of Washington School of Medicine, Seattle, Washington, USA; b Department of Molecular Microbiology and Immunology, Johns Hopkins Bloomberg School of Public Health, Baltimore, Maryland, USA

## Abstract

The time-honored mechanism of allocating funds based on ranking of proposals by scientific peer review is no longer effective, because review panels cannot accurately stratify proposals to identify the most meritorious ones. Bias has a major influence on funding decisions, and the impact of reviewer bias is magnified by low funding paylines. Despite more than a decade of funding crisis, there has been no fundamental reform in the mechanism for funding research. This essay explores the idea of awarding research funds on the basis of a modified lottery in which peer review is used to identify the most meritorious proposals, from which funded applications are selected by lottery. We suggest that a modified lottery for research fund allocation would have many advantages over the current system, including reducing bias and improving grantee diversity with regard to seniority, race, and gender.

**The lottery is in the business of selling people hope, and they do a great job of that.**

—John Oliver (1)

## EDITORIAL

The American research establishment has been facing the most prolonged funding crisis in its history. After a doubling in funding at the turn of the 20th century, the budget of the National Institutes of Health (NIH) was flat from 2003 to 2015, translating into a 25% reduction in actual buying power after taking inflation and the increasing costs of research into account (2). Although the increased NIH support in the 2016 spending bill is welcome news (3), this does not alter long-term uncertainty regarding the federal commitment to scientific research. The research funding crisis has been paralleled by other problems in science, including concerns about the reliability of the scientific literature, demographic imbalances, and various antiscience campaigns that question evolutionary theory, the usefulness of vaccines, human impact on climate change, and even the occurrence of the moon landings. What is perhaps most remarkable in this time of crisis and change is how little scientific leaders and governmental officials have done to combat these trends. Although each of these problems merits its own essay, we focus here on the allocation of U.S. biomedical research funds by the NIH. Specifically, we provide a detailed justification for the proposal that the NIH distribute funding through a modified lottery system, as briefly described in an Op-Ed in the *Wall Street Journal* last year (4).

## BIOMEDICAL RESEARCH FUNDING ALLOCATION IN THE UNITED STATES

The primary source of biomedical research funds in the United States is the NIH, which has an annual budget of approximately 30 billion dollars. The NIH-supported research enterprise consists of two groups: intramural researchers housed in NIH facilities and extramural investigators who are mostly housed in universities, medical schools, institutes, and industry. The ratio of funds spent on the intramural and extramural programs is roughly 1:10. In both cases, the allocation of funds is made according to peer review, but the NIH uses two very different mechanisms for assessing investigators. Intramural investigators are usually evaluated through retrospective peer review, where their recent accomplishments are used to make funding decisions, a mechanism similar to that used by the Howard Hughes Medical Institute. In contrast, funding allocations to the extramural program, which comprises the overwhelming majority of the NIH budget, is allocated by a mechanism of prospective peer review in which scientists must write grant proposals detailing future work that are reviewed and criticized by a panel of experts known as a study section. The difference in funding mechanisms used by the intramural and extramural programs is significant because it shows that there is already some flexibility in the approach used by the NIH to distribute its research dollars. In this essay, we will focus on the prospective peer review mechanism used to allocate funds to extramural investigators. The fundamentals of NIH extramural peer review have not changed in a half-century. The process involves writing a proposal that is reviewed by a panel of “peers” and assigned a priority score that is converted to a percentile ranking. The NIH then funds proposals depending on the amount of money available, with the payline being that percentile ranking up to which funding is possible. At the time that the system was designed, paylines exceeded 50% of the grant applications received. However, in recent decades there has been a precipitous drop in the proportion of grants that are funded. Today’s paylines and success rates are at historically low levels, hovering at around 10% in some institutes. Despite a drastic reduction in the likelihood of funding success, the essential features of NIH peer review and funding allocation have not changed.

## SHORTCOMINGS OF THE PRESENT SYSTEM

What is the desired product of scientific research? This question does not have a simple answer, but one measurable outcome is the generation of primary research publications, which are in turn cited by other publications. Remarkably, NIH study sections are unable to accurately predict which grant applications are likely to exhibit the highest publication productivity. Although a recent analysis of more than 130,000 NIH-funded grant applications reported a correlation between percentile scores and productivity (5), those findings contrast with several earlier studies showing poor predictive power for grant application peer review. Consequently, we reanalyzed the subset of the data for the grants awarded scores in the 20th percentile or better and found that the predictive ability of peer review was scarcely superior to what would be achieved by random chance and that differences in the median productivity exhibited by grants with high or low scores within this range were trivial (6). Our results corroborate earlier studies of more than 400 competing renewal R01 applications at the National Institute of General Medical Sciences (7) and 1,492 R01 applications at the National Heart, Lung, and Blood Institute (8). Hence, the available evidence makes a powerful case that the primary mechanism for biomedical research fund allocation in the United States is inadequate for prioritizing which applications to fund. The aforementioned analyses were preceded by studies suggesting that the NIH peer review process lacks statistical rigor. Only two to three reviewers in a typical study section carefully read an individual grant application and provide comments, and this reviewer sample size is too low to provide an acceptable level of precision (9). This criticism is not unique to the NIH, as studies from many countries have identified problems with the precision of grant peer review. In Canada, Mayo et al. found that the use of only two primary reviewers results in considerable randomness in funding decisions that could be improved by involving an entire 11-member review panel in the assessment of each application (10). Graves et al. examined variability in scores for the National Health and Medical Research Council of Australia and concluded that 59% of funded grants could miss funding simply on the basis of random variability in scoring (11). An analysis of applications to the Australian Research Council found interrater reliability for reviews to be poor (12), and researchers in Finland did not find that the reliability of grant peer review is improved by panel discussions (13). A French study observed that individual reviewers do not even tend to exhibit agreement on the weighting of criteria used for the grant review process (14).

A central weakness in the current system may be that experts are being asked to confidently predict the future of a scientific project, an inherently uncertain proposition. In this regard, the University of Pennsylvania psychologist Philip Tetlock showed that experts not only fared poorly in attempting to predict the future but also overrated their own abilities to do so (15). Another question is whether publication productivity is even the best metric on which to judge scientific success. Are study sections able to recognize potentially transformative research? Probably not, because intense competition for funding encourages both reviewers and applicants to be more cautious. The very structure of the NIH peer review system may encourage conformity and discourage innovation (16) of the type that could lead to scientific revolutions (17). As Nobel laureate Roger Kornberg has observed, “In the present climate especially, the funding decisions are ultraconservative. If the work that you propose to do isn’t virtually certain of success, then it won’t be funded. And of course, the kind of work that we would most like to see take place, which is groundbreaking and innovative, lies at the other extreme” (18). The NIH recognizes this problem and has created the Transformative Research Award Program, but of course, this does not solve the problem that transformative breakthroughs are often only evident as such after the fact (19).

There is also the critically important issue of bias. Sources of potential bias in peer review include cronyism and preference or disfavor for particular research areas, institutions, individual scientists, gender, or professional status. Reviewer bias can potentially have a major effect on the course of science and the career success of individual applicants. One meta-analysis of peer review studies found evidence of gender bias, such that women were approximately 7% less likely to obtain funding than men (20). Studies focusing specifically on the NIH have found comparable success in men and women submitting new R01 applications but lower success rates for women submitting renewal applications (21). There is also a continuing concern about racial bias in NIH peer review outcomes. Despite a number of initiatives following a study showing that black applicants were significantly less likely to be awarded NIH funding after controlling for educational background, country of origin, training, previous awards, publication record, and employer characteristics (22), as yet there is no evidence that the racial gap in funding success has improved (23). NIH peer reviewers tend to give better scores to applications closer to their area of expertise, and several studies have suggested that reviewers are influenced by direct or indirect personal relationships with an applicant (24).

The influence of grant reviewers in determining the fate of an application is directly proportional to the payline. This is an essential criticism of the current system, for it makes single individuals disproportionately powerful in their ability to influence the outcome of peer review. When generous paylines are available, applicants are likely to succeed even if there are scientific disagreements between applicants and/or reviewers. However, with shrinking paylines, the negative assessment by a single individual is often sufficient to derail a proposal. In this environment, a few individuals can profoundly influence the direction of research in an entire field. Reviewers are typically appointed for 4-year terms, allowing them to influence their fields for protracted periods of time. A Bayesian hierarchical statistical model applied to 18,959 R01 proposals scored by 14,041 reviewers found substantial evidence of reviewer bias that was estimated to impact approximately 25% of funding decisions (25). Day performed a computer simulation of peer review and found that very small amounts of bias can skew funding rates (25). This is not a new problem—in 1981, Cole et al. found that the odds of a proposal submitted to the National Science Foundation (NSF) getting funded were largely based on chance—the chance that specific reviewers would be chosen (26). “Targeting” research on the basis of program priorities can exacerbate the problem of bias and perversely lead to missed opportunities in basic research. The history of science is filled with stories of landmark discoveries by scientists who were looking for something else entirely—a third of anticancer drugs have been found by serendipity rather than by targeted cancer drug discovery research (27). Yet, funding agencies continue to attempt to target research funding to perceived priority areas, while support for undirected investigator-initiated projects has declined sharply (28).

Both applicants and reviewers have adapted to the funding crisis in ways that may be counterproductive to science. Applicants have responded by writing more grant applications, which takes time away from their research. As most applications are not funded, this largely represents futile effort. Some scientists estimate that half or more of their professional time is spent in seeking funding (29). In contrast, reviewers are asked to decide between seemingly equally meritorious applications and may respond by prioritizing them on the basis of “grantsmanship” (30), which has never been shown to correlate with research productivity or innovation. One of the most controversial aspects of NIH grant policy was the decision to limit applicants to two submissions of a research proposal (31). Under this policy, at a time when paylines were as low as 6%, many projects deemed meritorious by study sections were not only rejected but prohibited from resubmission for 37 months. With the current pace of science, this led to the death of many perfectly good ideas. Although this policy has now been modified to allow investigators to resubmit their projects as new grants (32), substantial damage has been done.

Peer review is used in both the ranking of grant applications and the evaluation of scientific papers. However, there are significant differences in how peer review of grant applications and papers operates. For grant applications, reviewers are chosen by an administrator who may or may not have in-depth knowledge of the relevant field, and review panels do not necessarily include the expertise necessary to review all proposals. For papers, reviewers are chosen by an editor who usually has expertise in the subject matter and can select reviewers with specific expertise in the subject area. Hence, a major difference between study section and manuscript peer review is that the latter is more likely to achieve a close match between subject matter and expertise. Accordingly, grant review is a more capricious process than manuscript review, and a single rogue reviewer can sink an application by assigning low scores without even needing to provide a convincing rationale for those scores. Publication decisions are made by editors, who can directly discuss areas of disagreement with authors and overrule single negative reviews at their discretion. Furthermore, authors have the option to appeal rejection decisions or submit their work to another journal. In contrast, there is no process for negotiation with scientific review administrators and little or no alternative to NIH funding. Another major difference is that the negative consequences of peer review differ for manuscript and grant applications, since the former usually find another publishing venue, whereas a denied grant application means that the proposed work cannot be done. Therefore, peer review of grant applications is of much greater importance for science than peer review of scientific manuscripts.

A critical aspect of the current crisis is that success rates for grant applications have fallen by more than two-thirds since the 1960s (33), and yet the system for fund allocation has essentially remained the same. A recent survey of researchers submitting proposals to the National Aeronautics and Space Administration (NASA), the NIH, and the NSF showed that even highly productive researchers are facing a 50% likelihood of not obtaining funding in the current cycle, resulting in the defunding of one-eighth of active programs following three such cycles (34). The authors of this survey estimated that at current funding rates, 78% of applicants will be unable to obtain federal funding for their research. This raises two obvious questions: (i) why has the system remained the same and (ii) why do scientists persist in this low-yield activity? Although we are not privy to discussions and decisions that have occurred among government leaders, it seems likely that the system has remained the same in the hope that national funding allocations will improve and because of the inertia involved in changing a mechanism that had worked relatively well for decades. As to why scientists persist in trying, the literature on the psychology of gambling behavior may provide some clues. People feeling desperate about their prospects will purchase lottery tickets as a surrogate for hope (35). Desperation is certainly prevalent in today’s scientific community (36). Entrapment in a system due to a previous investment of time and resources is also commonly invoked as an explanation for gambling (37), and many scientists have difficulty envisaging an alternative career path. In fact, current trends in science demand so much specialization (38) that most scientists are unable to shift into fields where funding may be more plentiful. Intelligence and a high level of executive function, as seen in most scientists, are correlated with susceptibility to maladaptive decision-making and the “gambler’s fallacy” (39). Risk-taking behavior may even have a neurological basis. Optimism has been described as a *sine qua non* for scientists (40), and irrational optimism correlates with reduced tracking of estimation errors by the right inferior prefrontal gyrus (41).

## PROPOSALS FOR REFORM

Recent systematic studies show that NIH grant peer review fails in its primary goal of stratifying meritorious applications when it comes to predicting the primary research outcome of citation metrics (6–8). Despite data to the contrary, the CSR (NIH Center for Scientific Review) continues to defend its methods (42). Recent reforms in NIH peer review have failed to address the inherent unfairness of the system (43). The NIH spends a lot of money on grant peer review. The annual budget of the CSR is $110 million, which pays for more than 24,000 scientists reviewing approximately 75,000 applications and attending approximately 2,500 panel meetings (42). The costs are not only economic. Writing and reviewing grants are extremely time-consuming and divert the efforts of scientists away from doing science itself. Specifically, the NIH is asking scientists who perform peer review to perform the impossible, e.g., discriminate among the best proposals, which results in arbitrary decisions, leads to psychological stress on both reviewers and applicants, and may not be funding the most important science. Recognizing the flaws in the current grant funding process, some scientists have suggested alternative approaches that would represent a radical departure from the present peer review system. Johan Bollen has suggested having scientists vote on who deserves funding (44). Michele Pagano recommends basing funding for established scientists on track record and a one-page summary of their plans (45). This approach has some empirical support, as prior publication productivity has been shown to correlate with future productivity of R01 grant recipients (46). John Ioannidis has proposed a number of options ranging from awarding small amounts of funding to all applicants to assigning grants randomly or basing awards on an applicant’s publication record (47). Recently, we proposed that the NIH adopt a hybrid approach based on a modified lottery system (4).

## LESSONS FROM THE WORLD OF FINANCE

The debate over the optimal strategy for allocating funds for scientific research has interesting parallels with the decisions involved in making financial investments. In 1973, the economist Burton Malkiel published his now-classic book, *A Random Walk down Wall Street* (48). Malkiel argued that investors cannot consistently outperform stock market averages, and therefore, a passive investment strategy can be just as effective as an active one. In fact, very few professional investors consistently outperform the market. A study called “Does Past Performance Matter?” by S&P Dow Jones found that only 2 out of 2,862 funds were able to remain in the top quarter over five successive years, worse than might be predicted by random chance alone—“If all of the managers of these mutual funds hadn’t bothered to try to pick stocks at all—if they had merely flipped coins—they would, as a group, probably have produced better numbers” (49). Even Warren Buffett has instructed in his will to “Put 10% in short-term government bonds and 90% in a very low-cost index fund … I believe the long-term results from this policy will be superior to those attained by most investors—whether pension funds, institutions, or individuals—who employ high-fee managers” (50). In 2007, the statistician Nassim Nicholas Taleb published the acclaimed book *The Black Swan* (51), which argued that the most influential events were both highly improbable and unpredictable. According to Taleb, investors should not attempt to predict such events but instead should construct a system that is sufficiently robust to withstand negative events and maximize the opportunity to benefit from positive ones. Applied to science, this suggests that it may be futile for reviewers to attempt to predict which grant applications will produce unanticipated transformational discoveries. In this regard, our recent review of revolutionary science suggests that historical scientific revolutions lack a common structure, with transformative discoveries emerging from puzzle solving, serendipity, inspiration, or a convergence of disparate observations (19). Consequently, a random strategy that distributes funding as broadly as possible may maximize the likelihood that such discoveries will occur. Taleb underscores the limits of human knowledge and cautions against relying on the authority of experts, emphasizing that explanations for phenomena are often possible only with hindsight, whereas people consistently fail in their attempts to accurately predict the future.

Four European economists have raised the question “Given incomplete knowledge of the market, is a random strategy as good as a targeted one?” (52, 53). A computer simulation was performed using data from British, Italian, German, and American stock indices. The authors compared four different conventional investment strategies with a random approach. Over the long run, each strategy performed similarly, but the random strategy turned out to be the least volatile, i.e., the least risky strategy with little compromise in performance. Given that assigning funds for investment or research allocation each involves a wager on future success with incomplete information, these lessons from the world of finance have relevance to science funding. Among the advantages of index funds are that randomization of the investment process can reduce “herding behavior” and financial “bubbles” (which raises the question of whether we are heading for microbiome and precision medicine “bubbles”—but that is a discussion for another time). An indexed strategy for picking stocks reduces the administrative costs associated with fund management, just as a modified lottery system for grant allocation could reduce the administrative costs of review.

## GOALS OF A FUNDING ALLOCATION SYSTEM

As we consider reform proposals for grant peer review, it is important to state some basic principles that we believe are likely to be accepted by the majority of scientists. First, we recognize that there are qualitative and quantitative differences among research proposals. Clearly, not all scientific projects are equally meritorious. We currently rely on the assessment of experts in the form of peer review to determine those differences. An ideal system would be a meritocracy that identified and funded the best science, but the available evidence suggests that the current process fails in this regard, and the goal might in fact be impossible. Second, we argue that some form of peer review will be required for funding allocation. Although we have catalogued many problems with the current peer review system, it is essential to have grant proposals evaluated by panels of scientists who have expertise in the area. Although experts may not be able to discriminate between meritorious proposals, they are still generally able to weed out proposals that are simply infeasible, are badly conceived, or fail to sufficiently advance science. Third, scarce research funds should be distributed in a fair and transparent manner. While fairness is likely to be partly in the eye of the beholder, there are mechanisms that are generally acknowledged to be fair. Specifically, there is a need to neutralize biases in funding decisions. Otherwise, the enormous power of reviewers at a time of unfavorable paylines will distort the course of science in certain fields. In this regard, there is evidence for increasing inefficiency in the translation of basic discovery into medical goods (54, 55). Although the causes for this phenomenon are undoubtedly complex, any bias in funding decisions affects the type of research done, which in turn influences potential downstream benefits for society. Should the review process favor new investigators? A case can certainly be made for the importance of providing support to new investigators, as they represent the future of science (56). This should not be taken to suggest that older investigators are unimportant. In fact, higher publication productivity has been seen for competing renewals than for new grants, and for projects directed by senior investigators (57). Nevertheless, we recognize that established investigators have significant advantages relative to new investigators with regard to experience, prior productivity, reputation in the field, and laboratories that are already established and productive. In a world of plentiful research funds, new investigators are able to compete successfully for funding with established laboratories. However, in times of funding scarcity, differences between established and new investigators can become magnified to favor established investigators over new ones. Established investigators benefit from the so-called “Matthew effect,” whereby those with resources and prestige are more likely to receive further rewards (58). Consequently, steps should be taken to improve the opportunities for new investigators as a matter of science planning policy. A modified lottery system could immediately benefit young investigators by creating a more level playing field.

## POTENTIAL BENEFITS OF A FUNDING LOTTERY

**FIG 1:**
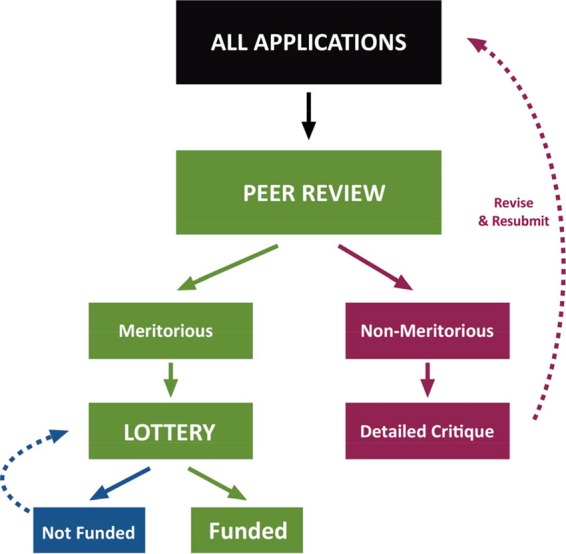
Proposed scheme for a modified funding lottery. In stage 1, applications are determined to be meritorious or nonmeritorious on the basis of conventional peer review. Nonmeritorious applications may be revised and resubmitted. In stage 2, meritorious applications are randomized by computer and funding is awarded to as many applications as funds permit on the basis of randomly generated priority scores.

Given overwhelming evidence that the current process of grant selection is neither fair nor efficient, we instead suggest a two-stage system in which (i) meritorious applications are identified by peer review and (ii) funding decisions are made on the basis of a computer-generated lottery (Fig. 1). The size of the meritorious pool could be adjusted according to the payline. For example, if the payline is 10%, then the size of the meritorious pool might be expected to include the top 20 to 30% of applications identified by peer review. This would eliminate or at least alleviate certain negative aspects of the current system, in particular, bias. Critiques would be issued only for grants that are considered nonmeritorious, eliminating the need for face-to-face study section meetings to argue over rankings, which would bring about immediate cost savings. Remote review would allow more reviewers with relevant expertise to participate in the process, and greater numbers of reviewers would improve precision. Funding would be awarded to as many computer-selected meritorious applications as the research budget allows. Applications that are not chosen would become eligible for the next drawing in 4 months, but individual researchers would be permitted to enter only one application per drawing, which would reduce the need to revise currently meritorious applications that are not funded and free scientists to do more research instead of rewriting grant applications. New investigators could compete in a separate lottery with a higher payline to ensure that a specific portion of funding is dedicated to this group or could be given increased representation in the regular lottery to improve their chances of funding. Although the proposed system could bring some cost savings, we emphasize that the primary advantage of a modified lottery would be to make the system fairer by eliminating sources of bias. The proposed system should improve research workforce diversity, as any female or underrepresented minority applicant who submits a meritorious application will have an equal chance of being awarded funding. There would also be benefits for research institutions. A modified lottery would allow research institutions to make more reliable financial forecasts, since the likelihood of future funding could be estimated from the percentage of their investigators whose applications qualify for the lottery. In the current system, administrators must deal with greater uncertainty, as funding decisions can be highly unpredictable. Furthermore, we note that program officers could still use selective pay mechanisms to fund individuals who consistently make the lottery but fail to receive funding or in the unlikely instance that important fields become underfunded due to the vagaries of luck.

The proposed system would treat new and competing renewal applications in the same manner. Historically, competing applications have enjoyed higher success rates than new applications, for reasons including that these applications are from established investigators with a track record of productivity. However, we find no compelling reason to justify supporting established programs over new programs.

Although we recognize that some scientists will cringe at the thought of allocating funds by lottery, the available evidence suggests that the system is already in essence a lottery without the benefits of being random (6). Furthermore, we note that lotteries are already used by society to make difficult decisions. Historically, a lottery was used in the draft for service in the armed forces. Today, lotteries are used to select students for charter schools (59), to determine the order of selection in the National Basketball Association draft, to issue green cards for permanent residency, and even to allocate scarce medical resources (60). Modified lotteries have been advocated as the fairest way in which to allocate scarce medical resources such as vaccines and organs for transplantation (61, 62). If lotteries could be used to select those who served in Vietnam, they can certainly be used to choose proposals for funding. We note that we are not the first to arrive at this idea (63). In fact, the New Zealand Health Research Council has already adopted a lottery system to select recipients of investigator-initiated Explorer grants (64).

The institution of a funding lottery would have many immediate advantages. First, it will maintain an important role for peer review at the front end, to decide which applications are technically sound enough to merit inclusion in the lottery. Second, it will convert the current system with its biases and arbitrariness into a more transparent process. Third, it will lessen the blow of grant rejection, since it is easier to rationalize bad luck than to feel that one failed to make the cut due to a lack of merit. Fourth, it will relieve reviewers from having to stratify the top applications, since it is increasingly obvious that this is not possible. Fifth, meritorious but unfunded proposals could continue to have a shot at receiving funding in the future instead of being relegated to the dustbin. Sixth, it will be less expensive to administer, and some of the funds currently used for the futile exercise of ranking proposals could be devoted instead to supporting actual scientific research. Seventh, it should decrease cronyism and bias against women, racial minorities, and new investigators. Eighth, it would give administrators in research institutions a greater capacity to make financial projections based on the percentage of their investigators who qualify for the lottery. Ninth, the system will be less noisy, will be fairer, and may promote new areas of investigation by removing favoritism for established fields that are better represented in review panels. Tenth, the realization that many meritorious projects remain unfunded may promote more serious efforts to improve research funding and study alternative approaches to peer review. In fact, the success rate of the lottery would provide a clear number for society and politicians to understand the degree to which meritorious research proposals remain unfunded, and this would hopefully lead to an increased budgetary allocation for research and development. Under the current system, the underfunding of science is hidden by the fallacious mantra that the most worthy science continues to be funded, which provides an excuse for inaction. A recent NSF report indicated that 68% of applications were rated as meritorious but only a third of these are funded (65).

## CONCLUDING REMARKS

The biologist E. O. Wilson has compared scientists to prospectors searching for gold (66): “In the 17th, 18th and 19th centuries, making scientific discoveries was like picking nuggets off the ground.” But, prospecting today is more challenging. The rewards are still great, but the big finds are more elusive. Targeted initiatives would direct all scientists to look for new lodes in the same place, while “transformative research” initiatives aim to fund only those who strike it rich. Neither strategy is optimal. Society must accept that science, as John Ioannidis has astutely observed, is an inherently “low-yield endeavor” (67). However, this low-yield endeavor has consistently improved the lot of humanity since the scientific revolution of the 17th century and remains humanity’s best bet for finding solutions to deal with such challenges as climate change, pandemics and disease, a faltering green revolution, and the need for new energy sources (68, 69). To continue to reap the maximal benefits of scientific exploration, researchers must be encouraged to search as far and wide as possible, leaving no stone unturned, even though only some will be successful in their quests. As Nassim Nicholas Taleb has written, “The reason markets work is because they allow people to be lucky, thanks to aggressive trial and error, not by giving rewards or incentives for skill” (51). We must provide our scientists with an opportunity to get lucky.
