# Erratum for Fang and Casadevall, Research Funding: the Case for a Modified Lottery

**DOI:** 10.1128/mBio.00694-16

**Published:** 2016-05-17

**Authors:** Ferric C. Fang, Arturo Casadevall

**Affiliations:** a Departments of Laboratory Medicine and Microbiology, University of Washington School of Medicine, Seattle, Washington, USA; b Department of Molecular Microbiology and Immunology, Johns Hopkins Bloomberg School of Public Health, Baltimore, Maryland, USA

## ERRATUM

Volume 7, no. 2, doi:10.1128/mBio.00422-16, 2016. On the second page of the article (PDF page 2), the sentence “A Bayesian hierarchical statistical model applied to 18,959 R01 proposals scored by 14,041 reviewers found substantial evidence of reviewer bias that was estimated to impact approximately 25% of funding decisions” cites the wrong reference. The correct reference should be as follows:

**Johnson VE.** 2008. Statistical analysis of the National Institutes of Health peer review system. Proc Natl Acad Sci U S A **105:**11076–11080. http://dx.doi.org/10.1073/pnas.0804538105. 

